# Socio-behavioral correlates of pre-exposure prophylaxis use and correct adherence in men who have sex with men in West Africa

**DOI:** 10.1186/s12889-022-14211-8

**Published:** 2022-09-29

**Authors:** August Eubanks, Bakary Coulibaly, Bintou Dembélé Keita, Camille Anoma, Ter Tiero Elias Dah, Ephrem Mensah, Sékou Kaba, Kpassou Julien Lokrou, Faïçal Rodrigue Ouedraogo, Alèda M. Fidèle Badjassim, Gwenaëlle Maradan, Michel Bourrelly, Marion Mora, Lucas Riegel, Daniela Rojas Castro, Issifou Yaya, Bruno Spire, Christian Laurent, Luis Sagaon-Teyssier, on behalf of the CohMSM-PrEP Study Group, Sayouba Ouedraogo, Bruno Granouillac, Laetitia Serrano, Martine Peeters, Cyril Berenger, Marion Fiorentino, Paméla Palvadeau, Bea Vuylsteke, Irith De Baetselier, Thijs Reyniers, Tania Crucitti, Fodié Diallo, Alou Coulibaly, Kader Maïga, Drissa Camara, Mahamadou Diarra, Aly Ouologuem, Abdoul Aziz Keita, Fodé Traoré, Oumar Cissé, Bréhima Abdrahamane Ouary, Ibrahima Kanta, Malan Jean-Baptiste Kouame, Rachelle Kotchi, Niamkey Thomas Aka, Noufo Hamed Coulibaly, Jean Armel Ekessi Koffi, Frédéric Dibi N’guessan, Stéphane-Alain Babo Yoro, Adama Cissé, Issa Traoré, Camille Rajaonarivelo, Joseph Ouedraogo, Juste Rodrigue Touré, Christian Coulibaly, Mamadou Ouedraogo, Elisabeth Thio, Ousseni Ilboudo, Abdoulazziz Traoré, Honoré Comsiambo, Richard Mawuényégan Kouamivi Agboyibor, Anani Attisso, Anouwarsadat Kokouba, Aléda Mawuli Badjassim, Kouakou Kokouvi Selom Agbomadji, Messan Attiogbe, Kossi Jeff Yaka, Agbégnigan Lorette Ekon, Julien Bimba, Claver Anoumou Yaotsè Dagnra

**Affiliations:** 1 Aix Marseille Univ, INSERM, IRD, SESSTIM, Sciences Economiques and Sociales de La Santé and Traitement de L’Information Médicale, ISSPAM, Marseille, France; 2 ARCAD Santé PLUS, Bamako, Mali; 3 Espace Confiance, Abidjan, Côte d’Ivoire; 4 Association African Solidarité, Ouagadougou, Burkina Faso; 5 Centre Muraz, Centre Muraz, Institut National de Santé Publique, Bobo-Dioulasso, Burkina Faso; 6 Espoir Vie Togo, Lomé, Togo; 7 ORS PACA, Observatoire Régional de La Santé Provence-Alpes-Côte d’Azur, Marseille, France; 8 Coalition Plus, Community-Based Research Laboratory, Pantin, France; 9 TransVIHMI, Univ Montpellier, INSERM, Montpellier, IRD France

**Keywords:** MSM, HIV, PrEP, Community-based research, West Africa

## Abstract

**Background:**

Multiple barriers compromise pre-exposure prophylaxis (PrEP) engagement (i.e., use and adherence) in men who have sex with men (MSM). In low/middle-income countries, little is known about PrEP engagement in this population. In West Africa, the CohMSM-PrEP study was one of the rare interventions providing PrEP to MSM. We estimated PrEP use and correct adherence rates in CohMSM-PrEP, together with associated factors over time.

**Methods:**

CohMSM-PrEP recruited MSM in four community-based clinics in Mali, Côte d’Ivoire, Burkina Faso, and Togo. Quarterly follow-up included collecting socio-behavioral data, and providing a comprehensive HIV prevention package, PrEP (daily or event-driven), and peer educator (PE)-led counselling. Using repeated measures, multivariate generalized estimating equations models were used to identify factors associated with self-reported i) PrEP use and ii) correct PrEP adherence during participants’ most recent anal intercourse (defined as four pills/week for daily users and 2 + 1 + 1 for event-driven users).

**Results:**

Five hundred twenty participants were included with a median follow-up time of 12 months (IQR 6–21). Of the 2839 intercourses declared over the follow-up period, PrEP use was self-reported for 1996 (70%), and correct PrEP adherence for 1461 (73%) of the latter. PrEP use was higher in participants who also attended participating clinics outside of scheduled visits (adjusted odds ratio (aOR) [95% Confidence Interval, CI], p-value; 1.32[1.01–1.71], 0.040), and in those who practiced condomless anal sex (1.86[1.54–2.24], < 0.001). Correct adherence was higher in those who often contacted PE outside of scheduled visits (2.16[1.01–4.64], 0.047) and in participants who adopted receptive/versatile sexual positions with stable partners (1.36[1.03–1.81], 0.030). Instead, after an interaction effect between financial situation and regimen was tested, it was lower in event-driven users with a difficult/very difficult financial situation (comfortable/just making ends meet & daily, 4.19[2.56–6.86], < 0.001; difficult/very difficult & daily, 6.47[4.05–10.30], < 0.001; comfortable/just making ends meet & event-driven, 1.63[1.22–2.17], 0.001), and in participants who felt alone (0.76[0.58–0.99], 0.042).

**Conclusions:**

Community-based clinic attendance and PE contact outside of scheduled visits were both associated with higher PrEP engagement, but some socially and economically marginalized participants struggled with adherence. As scale-up continues in West Africa, we recommend implementing community-based interventions and providing extra support for vulnerable users to ensure adequate PrEP engagement.

## Introduction

For men who have sex with men (MSM), multiple individual, social and structural barriers compromise pre-exposure prophylaxis (PrEP) engagement (i.e., use and correct adherence) [1–4], which can leave them exposed to HIV infection. While these barriers have been widely studied in high-income countries [5–19], very little is known about PrEP engagement in MSM in low- and middle-income countries, a setting where roll-out is comparatively limited [20–22] and where barriers to engagement can be exacerbated because of socially and culturally hostile contexts for MSM [23–25].

In West Africa, the HIV epidemic concentrated in MSM has persisted for over a decade. Prevalence in MSM is more than ten times higher (15.9%) than in the general population (1.2%) [26]. Although PrEP scale-up is urgent to contain the epidemic there, national programs in the region have been slow to implement it [27]. Structural barriers, economic constraints and biological factors disadvantage MSM and explain, in part, this concentrated epidemic and the delay in PrEP roll-out [28, 29]. Furthermore, legal barriers such as discriminatory policies and laws against homosexual behaviors reinforce a culture of widespread same-sex stigma [30–32]. At the institutional level, political stakeholders attribute less financial resources to HIV programs dedicated to LGBTQ populations [33], which limits the provision of adapted healthcare for MSM and PrEP scale-up for this population [28, 34–38]. These legal and financial barriers are compounded by heteronormative cultural values, which can lead to family rejection, social isolation and other forms of societal marginalization for MSM [30, 39]. Indeed, this complex legal and cultural context regarding MSM not only limits their access to tailored HIV prevention and care, including PrEP, but also research and clinical data on this population and their behaviors [29, 40–42], including PrEP engagement.

As in other regions, one of the biggest concerns for HIV prevention stakeholders in West Africa is poor PrEP effectiveness because of low PrEP use and adherence [43, 44]. In all income settings, individual level barriers such as the fear of side effects [5, 45–47], psychoactive substance use [5, 6], and low perceived risk of HIV infection [7–11] have all been associated with poorer PrEP-related outcomes and PrEP discontinuation. Also in all income settings, reporting higher levels of HIV risk behaviors has been associated with higher PrEP use and/or adherence [5, 6, 12–16, 48–50]. In low- and middle-income countries only, receiving partner, familial, and social support [51–55] was strongly associated with PrEP engagement, while in high-income settings having a friend [17] or partner [16] on PrEP was a facilitator. In all income settings, at the community level, medical mistrust because of experiences of homophobia, intersectional stigma [18, 23], PrEP-related stigma [24, 25, 51, 54, 56, 57], being perceived as HIV positive [53, 58], and considered at risk of HIV infection [54, 59], are all major psychosocial barriers to PrEP use and adherence by MSM. Finally, the effects of structural barriers on PrEP-related outcomes in different income settings worldwide, for example socioeconomic strain [6, 9, 16] and societal-level stigma [2, 19], have also been studied.

In places like West Africa, where there is the potential for multiple intersecting barriers to impact MSM PrEP engagement, community involvement and social network interventions could be the key to ensuring its success. To date, the impact of these types of interventions on PrEP use and/or adherence has only been quantitatively measured in studies focusing on marginalized MSM in the United States [17, 60–62]. Some of these studies involved multi-modal interventions, making it difficult to establish a clear link between these interventions and improved PrEP engagement.

One demonstration project contributing data on this topic in West Africa was CohMSM-PrEP [63]. MSM were provided with PrEP (daily and event-driven) and peer education in MSM-friendly community-based clinics. Initial findings showed that the introduction of PrEP and the use of peer-based outreach over time influenced the type of participant who enrolled in the study [64], and that PrEP uptake was associated with an 80% reduction in the risk of new HIV infections. However, it was also found that PrEP engagement decreased over time [65], and that event-driven PrEP users experiencing socioeconomic strain, as well as other socially vulnerable participants, had a higher risk of being ineffectively protected against HIV [66].

The aim of the present study was to report the rates of PrEP use and correct PrEP adherence (defined as four pills per week for daily users and 2 + 1 + 1 for event-driven users) in CohMSM-PrEP, as well as associated factors over time. We studied multiple cohort-related factors, including MSM-friendly clinic attendance and PE contact, and other sociobehavioral correlates of PrEP engagement. We also explored whether the correlates of PrEP use were similar to those of adherence. Our main goal was to better understand the cohort’s initial findings in a context where PrEP roll-out continues in national HIV/AIDS plans and programs in West Africa. To our knowledge, this is the first quantitative study to explore the correlates of PrEP engagement in MSM in Sub-Saharan Africa (SSA).

## Methods

### Study design

In November 2017, the CohMSM-PrEP prospective cohort study was initiated to assess the acceptability and feasibility of PrEP for MSM as part of a comprehensive sexual health prevention package in community-based clinics in West Africa (Burkina Faso, Côte d’Ivoire, Mali, and Togo). The four study sites were MSM-friendly clinics run by community-based organizations: Centre Oasis run by the Association African Solidarité (AAS) in Ouagadougou (Burkina Faso); Clinique Confiance, run by the association Espace Confiance in Abidjan (Côte d’Ivoire); Clinique de Santé Sexuelle des Halles run by the Association pour la Résilience des Communautés pour l’Accès au Développement et à la Santé – ARCAD Santé PLUS (formerly ARCAD-SIDA) in Bamako (Mali); and Centre Lucia, run by Espoir Vie Togo (EVT) in Lomé (Togo). Previously, in these same sites, the feasibility and acceptability of implementing HIV prevention and care services for MSM were studied in the CohMSM cohort [67]. Apart from PrEP, the comprehensive sexual health prevention package was the same in both cohort studies.

MSM were informed about the CohMSM-PrEP study at the clinics through a specific network of community-based organizations and peer educators (PE). CohMSM participants wishing to continue follow-up in CohMSM-PrEP (therefore wishing to take PrEP) and new ‘potential’ participants had to meet the same eligibility criteria (a comparison of the two cohorts has been previously described [64].

Participants were eligible if they were 18 years or older, HIV-negative (status confirmed at study enrollment), MSM (defined as reporting at least one episode of anal intercourse [insertive or receptive] with another man in the six months preceding enrollment), and reported any of the following HIV at-risk criteria: (i) non-virally suppressed seropositive sexual partner (male or female), (ii) condomless anal or vaginal sex with multiple partners in the previous six months, (iii) a history of sexually transmitted infection (STI) in the previous six months, (iv) post-exposure prophylaxis use in the previous six months, or (v) requesting PrEP.

Medical staff collected clinical data at each quarterly follow-up visit (scheduled or not), including PrEP regimen and quarterly HIV and STI testing results. Trained research assistants administered standardized face-to-face questionnaires at enrollment and every three months thereafter, which collected data on individual characteristics, sexual behaviors, psychosocial factors, substance use, and PrEP and condom use.

During these same quarterly follow-up visits, participants were provided free clinical examinations, PrEP (event-driven or daily), HIV testing, screening and treatment for other STI, condoms and lubricants, and tailored prevention counseling. The latter was provided by PE and focused on PrEP adherence, risk reduction strategies (including condom use, switching between regimens, abstaining from certain high-risk activities, and other risk-reduction strategies), encouraging testing, and program retention. Specific adherence-based counseling was provided monthly during the first three months and every three months thereafter. PrEP was prescribed as follows: daily (one pill per day) or event-driven (2 + 1 + 1 dosing; i.e., 2 pills between 2–24 h before sex [1 if PrEP taken the previous day] followed by 1 pill 24 h and another 48 h after the first pill[s]). At each follow-up visit, in concertation with study doctors and/or PE, participants could decide to switch PrEP strategies or stop PrEP (temporarily or permanently) depending on their needs. Participants were screened for HIV using national algorithms (Abbott Determine HIV 1/2 assay [Abbott Laboratories, Chiba, Japan] and, if the result was positive, SD Bioline HIV-1/2 3.0 [SD, Gyeonggi-do, South Korea] or First Response HIV-1/2 assay [Premier Medical Corporation, Mumbai, India]) and those diagnosed HIV positive during follow-up were invited to initiate antiretroviral treatment immediately.

### Study population

The analyses for the present study included CohMSM-PrEP participants enrolled between November 2017 and November 2020 who had at least one available questionnaire from M3 to M36, since there was no pill intake at baseline. We excluded data for participants who declared no male partners in the previous three months (stable or casual). As the study focused on PrEP-based HIV prevention, participants who seroconverted during follow-up were censored at their seroconversion date.

### Outcomes

The study’s two research outcomes were PrEP use and adherence during participants’ most recent anal intercourse (receptive and/or insertive) with male partners only (stable or casual), as self-reported in participants’ quarterly questionnaires.

Specifically, PrEP use was defined as declaring PrEP use alone or in combination with condoms, irrespective of adherence (= 1). No PrEP use was defined as declaring condom use only or neither PrEP nor condom use (= 0).

The adherence outcome measured whether PrEP adherence was ‘correct’ (= 1) or ‘incorrect’ (= 0). Adherence was considered correct for daily users, if they had taken at least four pills in the week before their most recent intercourse because the literature has shown that such levels confer protection against HIV infection for MSM [50, 68]. For event-driven users, it was considered correct if they had taken PrEP as prescribed (2 + 1 + 1) [69, 70]. Incorrect PrEP adherence was defined as all other pill taking combinations or taking any pills before or after sex. Only measures of most recent intercourse where PrEP use was declared were included for this outcome.

### Covariates

Covariates in the present analysis included:

- *Sociodemographic and socioeconomic characteristics.* Age (continuous), country-fixed effects (Mali, Burkina Faso, Togo and Côte d’Ivoire), employment status (employed vs unemployed), perception of financial situation (‘comfortable’ and ‘just making ends meet’ vs ‘difficult’ and ‘very difficult’).
- *Cohort or PrEP-related characteristics.* Recruitment type (ex-CohMSM participant vs. new participant), chosen PrEP regimen (event-driven or daily), PrEP use (‘easy’ and ‘very easy’ vs ‘difficult’ and ‘very difficult’), attended clinic outside of scheduled visits (‘yes’ vs ‘no’), contacted a peer educator outside of scheduled visits (‘never,’ ‘sometimes’ [i.e., 4 or less times a month] ‘often’ [i.e., 2 or more times a week]), and follow-up time (3–6 months, 7–12 months, > 12 months).
- *MSM identity and psychosocial aspects.* Self-defined sexual orientation (‘heterosexual’, ‘homosexual/gay/trans [i.e., transsexual or transgender],’ or ‘bisexual’), self-defined gender identity (‘man/boy’ vs ‘both a man and a woman,’ ‘more a woman,’ and ‘neither a woman nor a man’), overall perceived homosexuality stigma score (based on 11 homosexuality-related stigma questions where overall low (high) stigma was defined as < median (vs ≥ median) [71]), and feeling alone (‘no’ vs ‘sometimes’ and ‘every day’).
- *Sexual behaviors and substance use.* Recreational drug use in the previous month (no use vs used at least one of the following: cannabis/marijuana/hash/weed, Tramadol, cocaine/crack, heroin/other opioids, medicines used for recreational purposes, Kenacort/other injectable skin lightening products, other), condomless anal sex (CAS) during most recent intercourse (‘yes’ vs ‘no’), a receptive position during most recent anal intercourse (‘yes’ vs ‘no’), transactional sex in the previous three months (‘never’ vs ‘rarely,’ ‘often’ and ‘always’ vs ‘not concerned’), number of sexual intercourses with stable and/or casual partners in the previous month (categorized into ‘none or no partner’ vs ‘1–4’ vs ‘ ≥ 5’) and sexual position with stable male partner in the previous three months (‘no stable partner’ vs ‘exclusively insertive’ vs. ‘receptive or versatile’).

### Statistical analyses

The generalized estimating equation (GEE) method, using repeated measures and a binary logistic distribution function, was used to identify factors associated with PrEP use first, and then factors associated with correct PrEP adherence. For both outcomes, all univariate and multivariate models were adjusted for country-fixed effects and other confounders. All covariates with a p-value of 0.20 or less in the univariate analysis were eligible for the multivariate model. In line with the present study’s objectives, specific interactions between covariates were tested for during multivariate model specification, including PrEP regimen and perception of financial situation. The forward selection technique was used to construct the final multivariate model. The Quasi-likelihood Information Criterion (QIC) was used to verify the goodness-of-fit of the chosen model. All analyses were performed using STATA 14.0 statistical software.

### Ethical considerations

The study protocol was approved by the national ethics committees of Togo (N°338/2017/MSPS/CAB/SG/DGAS/DPML/CBRS), Mali (N°2017/113/CE/FMPOS), Burkina Faso (N°2017–7-105), and Côte d’Ivoire (N°088/MSHP/CNER-kp). All participants provided written informed consent.

## Results

### Sample characteristics

**Fig. 1:**
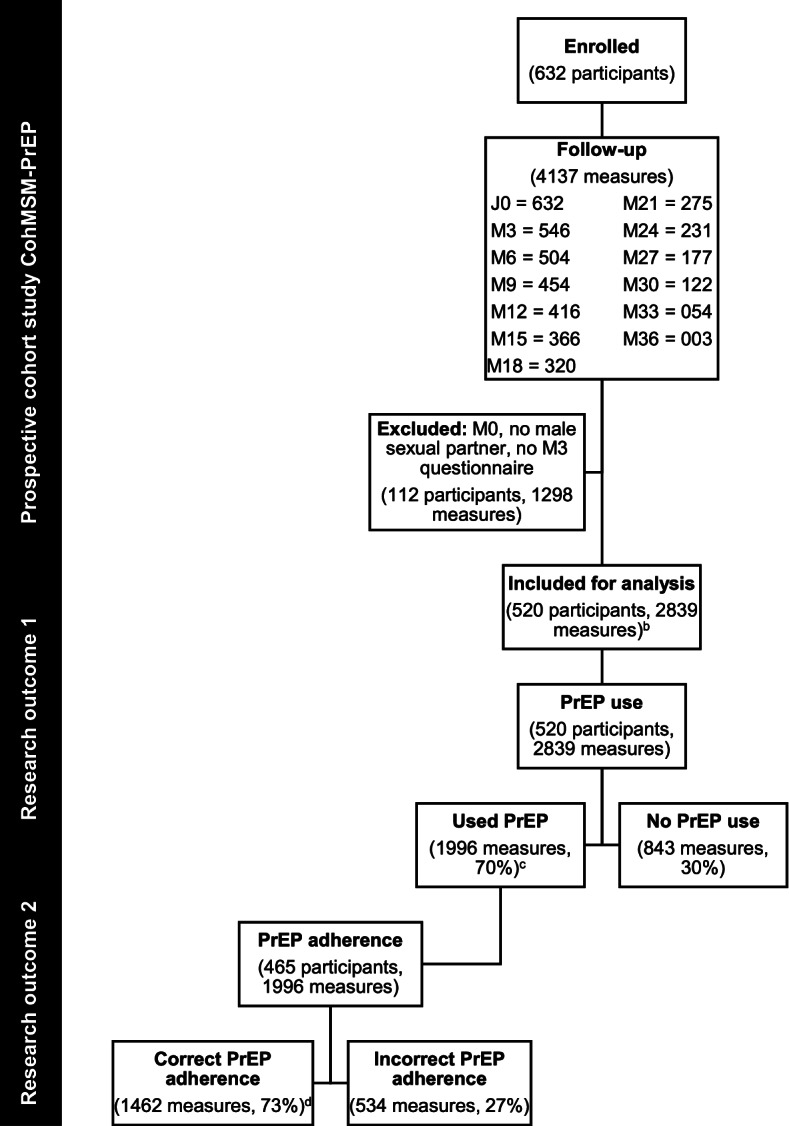
CohMSM-PrEP study population flowchart with research outcomes: i) PrEP use and ii) PrEP adherence^a^. ^a^During most recent anal intercourse with a male sexual partner; follow-up from November 2017-November 2020 (M0-M36); Median follow-up time = 12 months, IQR [6–21]. ^b^Present study population by PrEP regimen: 26.9% (765/2839) daily, 72.9% (2069/2839) event-driven and 0.2% (5/2839) missing. ^c^PrEP use by PrEP regimen: 79.1%, (605/765) of daily users and 67.0% (1386/2069) of event-driven users. ^d^Correct adherence by PrEP regimen: 88.8% (537/605) of daily users and 66.7% (925/1386) event-driven users. PrEP: pre-exposure prophylaxis

Of the 632 CohMSM-PrEP participants enrolled between November 2017 and November 2020, 520 (82%) had at least one available questionnaire from M3 to M36 (2839 observations), and were included in the present study (Fig. 1). Excluded participants (*n* = 112) either did not have a male sexual partner in the previous three months or had not yet received their M3 follow-up questionnaire.

After a median follow-up time of 12 months (IQR 6–21), of the 2839 most recent anal intercourses declared over the follow-up period, PrEP use (outcome 1) was declared for 1996 (70%) (Fig. 1). The remaining 843 intercourses (30%) were not protected by PrEP and were excluded from the PrEP adherence (outcome 2) assessment. Correct PrEP adherence accounted for 1461 (73%) of the 1996 intercourses where PrEP was used.

Of the 2839 most recent anal intercourses over the follow-up period, 26.9% (765/2839) concerned participants on daily PrEP, 72.9% (2069/2839) concerned participants on event-driven PrEP, and 0.2% (5/2839) had missing regimen data (Fig. 1). Four-fifths of daily users declared PrEP use during their most recent anal intercourse (79.1%, 605/765), compared to 67.0% (1386/2069) of event-driven users. When PrEP was used, 88.8% (537/605) of daily users declared correct adherence, compared to 66.7% (925/1386) of event-driven users.

Table 1 describes the characteristics of the entire study sample at baseline. Mean age was 25.6 years (standard deviation, SD = 5.8). Most participants (39.8%, 207/520) came from Mali, 23.2% (121/520) from Togo, 18.5% (96/520) Burkina Faso, and 18.5% (96/520) Cote d’Ivoire. Half (50%, 260/520) were employed, and 56.2% (292/520) perceived their financial situation as difficult/very difficult at baseline.

**Table 1:** Characteristics of study participants at baseline *N* = 520

Variable	n (%) or mean (SD)
Age^b^ (in years)	25.6 (5.8)
Country of inclusion	
Burkina Faso	96 (18.5)
Cote d’Ivoire	96 (18.5)
Mali	207 (39.8)
Togo	121 (23.2)
Employment status^c^	
Employed	260 (50.0)
Unemployed	252 (48.5)
Perception of financial situation^c^	
Difficult or very difficult	292 (56.2)
Comfortable or just making ends meet	220 (42.3)
Recruitment type	
Ex-CohMSM participant	290 (55.8)
New participant	230 (44.2)
Chosen PrEP regimen^ad^	
Event-driven	389 (74.8)
Daily	129 (24.8)
Using PrEP is^ae^	
Difficult or very difficult	68 (13.1)
Easy or very easy	432 (83.1)
Attended clinic outside of scheduled visits	
Yes	205 (39.4)
No	315 (60.6)
Contacted PE outside of scheduled visits^f^	
Never	355 (68.3)
Sometimes	143 (27.5)
Often	13 (2.5)
Self-defined sexual orientation^c^	
Heterosexual	14 (2.7)
Homosexual. gay. trans	202 (38.8)
Bisexual	296 (56.9)
Self-defined gender identity^c^	
Man or boy	307 (59.0)
Both a man and a woman; more a woman; neither a woman or a man	205 (39.4)
Perceived homosexuality stigma score^f^	
Low (< median)	251 (48.3)
High (≥ median)	217 (41.7)
Feeling alone^h^	
Yes	290 (55.8)
No	176 (33.8)
Recreational drug use in the previous month^h^	
Yes	53 (10.2)
No	413 (79.4)
Condomless anal sex during most recent intercourse^i^	
Yes	162 (31.2)
No	348 (66.9)
Receptive position during most recent anal intercourse^j^	
Yes	258 (49.6)
No	261 (50.2)
Transactional sex in the previous three months	
Never	219 (42.1)
Rarely. often. always	99 (19.0)
Not concerned	202 (38.9)
Number of sexual intercourses with stable partner in previous month^k^	
None or no stable partner	229 (44.0)
1–4	244 (46.9)
5 +	43 (8.3)
Number of sexual intercourses with casual partner(s) in previous month^c^	
None or no casual partner	254 (48.8)
1–4	228 (43.8)
5 +	36 (6.9)
Sexual position with stable male partner in the previous three months^l^	
No stable male partner	152 (29.2)
Exclusively inserted	146 (28.1)
Receptive or versatile	209 (40.2)

*SD* Standard deviation, *CohMSM* Cohort of MSM, *PrEP* Pre-exposure prophylaxis, *PE* Peer educator

^a^PrEP-related outcomes were only available from the M3 follow-up questionnaire since there was no pill intake at M0

^b^Range = 18 – 57, 11 (2.1%) missing values

^c^8 (1.5%) missing values

^d^2 (0.4%) missing values

^e^20 (3.8%) missing values

^f^9 (1.7%) missing values

^g^52 (10.0%) missing values

^h^54 (10.4%) missing values

^i^10 (1.9%) missing values

^j^1 (0.2%) missing values

^k^4 (0.8%) missing values

^l^13 (2.5%) missing values

Ex-CohMSM participants comprised 55.8% (290/520) of the study sample. Two-fifths (39.4%, 205/520) of the sample attended their study clinic outside of scheduled visits. In terms of contacting PE outside of scheduled visits, 68.3% (355/520) never contacted them, 27.5% (143/520) contacted them sometimes, and 2.5% (13/520) often. Most participants chose event-driven PrEP (74.8%, 389/520), and most (83.1%, 432/520) found it easy/very easy to use PrEP.

At baseline, most of the study sample self-identified as bisexual (56.9%, 296/520), followed by homosexual/gay/trans (38.8%, 202/520), and 2.7% heterosexual (14/520). Fifty-nine percent (307/520) self-identified as male, and 39.4% (205/520) as both a man and a woman, more a woman, or neither a woman nor a man. Approximately half (48.3%, 251/520)) of the participants had a low perceived homosexuality stigma score, while 41.7% (217/520) had a high score. Over half (55.8%, 290/520) felt alone at baseline and 10.2% (53/520) had used recreational drugs in the previous month.

With regard to stable male partners, 44% (229/520) of the participants had no stable male partner at baseline or no sexual intercourse with their partner in the previous month, while 46.9% (244/520) and 8.3% (43/520) had 1–4 and 5 + intercourses, respectively. With respect to casual partners, 48.8% (254/520) had no partner or no sexual intercourse in the previous month, while 43.8% (228/520) and 6.9% (36/520) had 1–4 and 5 + intercourses, respectively. In terms of intercourse with stable partners only in the previous three months, most participants had receptive or versatile intercourse (40.2%, 209/520), 28.1% (146/520) reported exclusively insertive intercourse, and 29.2% (152/520) were not concerned as they had no stable partner. Casual transactional sex in the previous three months was reported by 19% (99/520) of participants, while 42.1% (219/520) did not report it, and 38.9% (202/520) were not concerned as they did not have casual partners. During their most recent anal intercourse with either partner type, 31.2% (162/520) of the study sample had CAS, and almost half (49.6%, 258/520)) of these intercourses were receptive.

### PrEP use: univariate and multivariate analyses

Table 2 shows the univariate analysis for PrEP use (outcome 1). In univariate analysis, PrEP use during the most recent intercourse was more likely in daily PrEP users (*p* = 0.011), participants attending their clinic outside of scheduled visits (*p* = 0.037), and recent recreational drug users (*p* = 0.045). It was also more likely in those who had CAS (*p* < 0.001), those declaring receptive anal intercourse (*p* < 0.001), and having sexual intercourse with a stable male partner in the previous month (1–4, *p* < 0.001; 5 + , *p* = 0.003). Furthermore, when an interaction effect between perceived financial situation and PrEP regimen was tested, daily users with a comfortable or just making ends meet situation were more likely to declare PrEP use (*p* = 0.036). In contrast, being followed up for more than 12 months (*p* = 0.001), finding PrEP difficult or very difficult to use (*p* < 0.001), and self-identifying as male (*p* = 0.014) were negatively associated with PrEP use. Finally, a positive trend existed between PrEP use and often contacting PE outside of scheduled visits (*p* = 0.094), recent transactional sex and PrEP use (*p* = 0.081), and daily users with a difficult or very difficult financial situation (*p* = 0.085), while a negative trend existed between PrEP use and having had 5 + sexual intercourses with casual male partners in the previous month (*p* = 0.078).

**Table 2:** Univariate and multivariate analyses of factors associated with PrEP use (*n* = 520, 2839 measures)^a^ and with correct PrEP adherence (*n* = 465, 1995 measures)^b^

Variables	PrEP use			PrEP adherence		
	**n(%)**^c^	**Univariate**	**Multivariate**	**n(%)**^d^	**Univariate**	**Multivariate**
		**OR[95% CI], *p*-value**	**OR [95% CI], *p*-value**		**OR [95% CI], *p-*value**	**OR [95% CI], *p*-value**
Age		1.01 [0.99–1.03], 0.528	1.01 [0.99–1.03], 0.448		1.02 [1.00–1.05], 0.043	
Country of inclusion						
Burkina Faso	344 (17.2)	0.82 [0.57–1.16], 0.261	0.89 [0.60–1.30], 0.532	246 (16.8)	0.84 [0.57–1.24], 0.386	0.71 [0.46–1.09], 0.116
Cote d’Ivoire	250 (12.5)	0.46 [0.33–0.65], < 0.001	0.42 [0.29–0.62], < 0.001	162 (11.1)	0.56 [0.37–0.84], 0.006	0.45 [0.28–0.71], 0.001
Togo	558 (28.0)	0.85 [0.62–1.17], 0.327	0.70 [0.50–0.99], 0.045	418 (28.6)	1.00 [0.70–1.42], 0.997	1.02 [0.69–1.49], 0.937
Mali	844 (42.3)	1 (ref)	1 (ref)	636 (43.5)	1 (ref)	1 (ref)
Employment status						
Employed	1176 (58.9)	1.12 [0.90–1.38], 0.316		881 (60.3)	1.09 [0.84–1.40], 0.503	
Unemployed	802 (40.2)	1 (ref)		570 (39.0)	1 (ref)	
Perception of financial situation						
Comfortable or just making ends meet	928 (46.5)	0.99 [0.80–1.21], 0.892		750 (51.3)	1.27 [0.99–1.62], 0.050	
Difficult or very difficult	1050 (52.6)	1 (ref)		701 (47.9)	1 (ref)	
Perception of financial situation & PrEP regimen type						
Comfortable or just making ends meet & Daily	249 (12.5)	1.48 [1.03–2.13], 0.036		221 (15.1)	4.87 [3.00–7.89], < 0.001	4.19 [2.56–6.86], < 0.001
Difficult or very difficult & Daily	354 (17.7)	1.31 [0.96–1.79], 0.085		314 (21.5)	5.53 [3.54–8.63], < 0.001	6.47 [4.05–10.30], < 0.001
Comfortable or just making ends meet & Event-driven	676 (33.9)	0.98 [0.77–1.23], 0.839		480 (32.8)	1.58 [1.20–2.08], 0.001	1.63 [1.22–2.17], 0.001
Difficult or very difficult & Event-driven	694 (34.8)	1 (ref)		436 (29.8)	1 (ref)	1 (ref)
Follow-up time						
> 12 months	933 (46.7)	0.71 [0.58–0.86], 0.001		735 (50.3)	1.69 [1.36–2.11], < 0.001	1.80 [1.42–2.28], < 0.001
7–12 months	479 (24.0)	0.91 [0.75–1.11], 0.345		343 (23.5)	1.36 [1.09–1.70], 0.007	1.48 [1.14–1.92], 0.003
0–6 months	584 (29.3)	1 (ref)		384 (26.3)	1 (ref)	1 (ref)
Recruitment type						
Ex-CohMSM participant	1279 (64.1)	0.83 [0.64–1.07], 0.151	0.74 [0.56–0.98], 0.034	938 (64.2)	0.90 [0.68–1.21], 0.503	1.21 [0.88–1.65], 0.243
New participant	717 (35.9)	1 (ref)	1 (ref)	524 (35.8)	1 (ref)	1 (ref)
Chosen PrEP regimen						
Daily	605 (30.3)	1.38 [1.08–1.77], 0.011		925 (63.3)	4.21[2.97–5.96], 0.000	
Event-driven	1386 (69.4)	1 (ref)		537 (36.7)	1 (ref)	
Using PrEP is						
Difficult or very difficult	166 (08.3)	0.62 [0.49–0.78], < 0.001	0.64 [0.51–0.82], < 0.001	101 (6.9)	0.69 [0.50–0.95], 0.025	
Easy or very easy	1829 (91.6)	1 (ref)		1360 (93.0)	1 (ref)	
Attended clinic outside of scheduled visits						
Yes	1058 (53.0)	1.30 [1.02–1.68], 0.037	1.32 [1.01–1.71], 0.040	667 (45.6)	0.80 [0.61–1.06], 0.123	
No	938 (47.0)	1 (ref)	1 (ref)	795 (54.4)	1 (ref)	
Contacted PE outside of scheduled visits						
Often	61 (03.1)	1.69 [0.91–3.12], 0.094		46 (3.2)	1.55 [0.78–3.11], 0.214	2.16 [1.01–4.64], 0.047
Sometimes	618 (31.0)	1.01 [0.82–1.25], 0.901		427 (29.2)	0.79 [0.62–1.01], 0.060	0.82 [0.63–1.07], 0.140
Never	1298 (65.1)	1 (ref)		977 (66.8)	1 (ref)	1 (ref)
Self-defined sexual orientation						
Heterosexual	37 (1.9)	1.24 [0.61–2.54], 0.552		24 (1.6)	0.95 [0.43–2.07], 0.891	
Homosexual. gay. trans	818 (41.0)	0.97 [0.78–1.21], 0.820		608 (41.6)	1.13 [0.87–1.47], 0.364	
Bisexual	1123 (56.3)	1 (ref)		819 (56.0)	1 (ref)	
Self-defined gender identity						
Man or boy	1186 (59.4)	0.76 [0.61–0.94], 0.014	0.74 [0.59–0.94], 0.012	870 (59.5)	0.97 [0.75–1.24], 0.789	0.89 [0.67–1.19], 0.445
Both a man and a woman; more a woman; neither a woman nor a man	792 (39.7)	1 (ref)	1 (ref)	581 (39.7)	1 (ref)	1 (ref)
Perceived homosexuality stigma score						
Low (< median)	967 (48.5)	0.88 [0.71–1.09], 0.246		726 (49.7)	1.25 [0.97–1.61], 0.082	
High (≥ median)	966 (48.4)	1 (ref)		688 (47.1)	1 (ref)	
Feeling alone						
Yes	1042 (52.2)	0.94 [0.77–1.16], 0.572		745 (51.0)	0.76 [0.60–0.98], 0.032	0.76 [0.58–0.99], 0.042
No	865 (43.4)	1 (ref)		650 (44.5)	1 (ref)	1 (ref)
Recreational drug use in the previous month						
Yes	184 (9.2)	1.47 [1.01–2.14], 0.045	1.49 [1.01–2.19], 0.044	136 (9.3)	1.12 [0.73–1.72], 0.597	
No	1723 (86.3)	1 (ref)	1 (ref)	1259 (86.1)	1 (ref)	
Condomless anal sex during most recent intercourse						
Yes	843 (42.3)	1.92 [1.60–2.29], < 0.001	1.86 [1.54–2.24], < 0.001	612 (41.9)	0.93 [0.75–1.15], 0.503	
No	1153 (57.7)	1 (ref)	1 (ref)	850 (58.1)	1 (ref)	
Receptive position during most recent anal intercourse						
Yes	1024 (51.3)	1.48 [1.21–1.82], < 0.001		757 (51.8)	1.20 [0.94–1.52], 0.142	
No	972 (48.7)	1 (ref)		705 (48.2)	1 (ref)	
Transactional sex in the previous three months						
Not concerned	463 (23.2)	1.19 [0.90–1.59], 0.228		350 (23.9)	1.15 [0.83–1.61], 0.396	
Rarely, often, always	445 (22.3)	1.24 [0.97–1.56], 0.081		332 (22.7)	1.11 [0.85–1.47], 0.442	
Never	1088 (54.5)	1 (ref)		780 (53.4)	1 (ref)	
Number of sexual intercourses with stable partner in previous month						
5 +	168 (8.4)	1.59 [1.17–2.17], 0.003	1.50 [1.08–2.07], 0.014	113 (7.7)	0.74 [0.52–1.04], 0.086	
1–4	1158 (58.0)	1.51 [1.29–1.78], < 0.001	1.45 [1.23–1.72], < 0.001	870 (59.5)	1.07 [0.87–1.31], 0.540	
None or no stable partner	662 (33.2)	1 (ref)	1 (ref)	476 (32.6)	1 (ref)	
Number of sexual intercourses with casual partner(s) in previous month						
5 +	104 (5.2)	0.73 [0.51–1.04], 0.078		66 (4.5)	0.65 [0.43–0.99], 0.046	0.56 [0.34–0.92], 0.022
1–4	730 (36.6)	0.87 [0.73–1.03], 0.109		527 (36.1)	0.99 [0.80–1.23], 0.941	0.98 [0.76–1.28], 0.904
None or no casual partner	1159 (58.1)	1 (ref)		866 (59.2)	1 (ref)	
Sexual position with stable partner in the previous three months						
No stable partner	371 (18.6)	0.88 [0.70–1.10], 0.267		268 (18.3)	1.13 [0.85–1.49], 0.411	1.09 [0.78–1.51], 0.620
Receptive or versatile	894 (44.8)	1.10 [0.90–1.36], 0.350		675 (46.2)	1.30 [1.02–1.67], 0.033	1.36 [1.03–1.81], 0.030
Exclusively insertive	707 (35.4)	1 (ref)		503 (34.4)	1 (ref)	1 (ref)

*GEE* Generalized estimating equation, *OR* Odds ratio, *aOR* Adjusted odds ratio, *CI* Confidence interval, *ref* reference, *CohMSM* Cohort of MSM, *PE* Peer educator

^a^GEE, binary logistic distribution; adjusted for country-fixed effects, age and recruitment type, QIC = 3138

^b^GEE, binary logistic distribution; adjusted for country-fixed effects, recruitment type and self-defined gender identity, QIC = 2014

^c^PrEP use accounted for 1996 measures; no PrEP use accounted for 843 measures; the difference between the total sample and the total number of measures for each variable corresponds to missing values

^d^Correct PrEP adherence accounted for 1462 measures; incorrect PrEP adherence accounted for 534 measures; the difference between the total sample and the total number of measures for each variable corresponds to missing values

The results from the multivariate analysis of factors associated with PrEP use are also shown in Table 2. Age and recruitment type were confounders in the multivariate model specification and were therefore adjusted for in the final multivariate model in addition to country-fixed effects. PrEP use was more likely in the following participants: (i) those who attended the clinic outside of scheduled visits (Adjusted Odds Ratio, aOR [95% Confidence Interval, CI], *p*-value; 1.32 [1.01–1.71], 0.040), (ii) recent recreational drug users (1.49 [1.01–2.19], 0.044), (iii) participants who had CAS during their most recent sexual intercourse (1.86 [1.54–2.24], < 0.001), (iv) participants who had 1–4 intercourses with their stable partner in the previous month (1.45 [1.23–1.72], < 0.001) and (v) those who had 5 + intercourses with their stable partner in the previous month (1.50 [1.08–2.07], 0.014). Instead, PrEP use was less likely in participants who found it difficult or very difficult to use PrEP (0.64 [0.51–0.82], < 0.001) and those who self-identified as male (0.74 [0.59–0.94], 0.012).

### PrEP adherence: univariate and multivariate analyses

Table 2 also shows the univariate analysis for PrEP adherence (outcome 2). As participants’ age (*p* = 0.043) increased, so did the likelihood of having correct PrEP adherence. Correct adherence was also more likely in participants with 7–12 (*p* = 0.007) and > 12 months of follow-up (*p* < 0.001), in daily PrEP users (*p* < 0.001) as well as in those declaring a receptive or versatile sexual position with their stable male partner in the previous three months (*p* = 0.033). Instead, correct PrEP adherence was less likely in participants who found it difficult or very difficult to use PrEP (*p* = 0.025), those who felt alone (*p* = 0.032), and those who had 5 + sexual intercourses with a casual partner in the previous month (*p* = 0.046). Furthermore, when an interaction effect between perceived financial situation and PrEP regimen was tested, event-driven users with a difficult or very difficult financial situation were also less likely to have correct adherence (daily PrEP users with a comfortable financial situation or just making ends meet, *p* < 0.001; daily PrEP users with a difficult or very difficult financial situation, *p* < 0.001; and event-driven users with a comfortable financial situation or just making ends meet, *p* = 0.001). A negative trend was observed between correct PrEP adherence and having 5 + sexual intercourses with stable male partners (*p* = 0.086), as well as between correct adherence and a comfortable or just making ends meet financial situation (*p* = 0.050), while a positive trend was seen between correct adherence and high levels of PE contact (*p* = 0.060) and a low perceived homosexuality stigma score (*p* = 0.082).

Multivariate results are also shown in Table 2. Self-defined gender identity and recruitment type were confounders during the multivariate model specification, and were therefore adjusted for in the final multivariate model in addition to country-fixed effects. Correct PrEP adherence was more likely in the following participants: (i) participants who were followed for 7–12 months (1.48 [1.14–1.92], 0.003), (ii) those followed for more than 12 months (1.80 [1.42–2.28], < 0.001), (iii) participants who often contacted PE outside of scheduled visits (2.16 [1.01–4.64], 0.047), and (iv) those declaring a receptive or versatile sexual position with stable partners (1.36 [1.03–1.81], 0.030). Instead, correct PrEP adherence was less likely in participants who felt alone (0.76 [0.58–0.99], 0.042), those who declared 5 + intercourses with casual partners (0.56 [0.34–0.92], 0.022), and in event-driven users with a difficult or very difficult financial situation (daily PrEP users with a comfortable/just making ends meet financial situation, 4.19 [2.56–6.86], < 0.001; daily users with a difficult/very difficult situation, 6.47 [4.05–10.30], < 0.001; and event-driven users with a comfortable/just making ends meet situation, 1.63 [1.22–2.17], 0.001).

## Discussion

In the present study on MSM participants enrolled from 2017 to 2020 in the CohMSM-PrEP study in West Africa, of the 2839 most recent anal intercourses declared, PrEP use was self-reported for 1996 (70%), and correct PrEP adherence for 1461 of the latter (73%). These two PrEP engagement outcomes were mainly linked to cohort-related characteristics and participants’ sexual behaviors. In contrast, only PrEP adherence was associated with participants’ social and economic vulnerabilities. Understanding the factors separately associated with PrEP use and adherence will help guide PrEP rollout as it becomes more widely available, as it is important to understand not only who uses PrEP, but also who uses it correctly.

One of the most important results from the present study was the effect of certain cohort-related characteristics on both outcomes. Specifically, we found that attending participating MSM-friendly clinics outside of scheduled visits and contacting PE outside of scheduled visits were associated with PrEP use and correct adherence, respectively. In terms of other studies, a lack of MSM-friendly clinics is a known barrier to accessing HIV prevention services [72–74]. One important aspect of MSM-friendly clinics is community engagement, for example the use of PE. Studies conducted prior to the PrEP era, showed that community engagement and social network interventions improved MSM access to HIV services, and HIV prevention, care and risk outcomes [1, 75–77]. However, few data exist on the effect of these community-based aspects on PrEP engagement in MSM, especially in West Africa and SSA as a whole. Involvement in the MSM community has previously been shown to play a crucial role in effective HIV protection, through correct PrEP adherence and/or condom use in the CohMSM-PrEP cohort [66], and through protective PrEP concentrations in the iPrEx OLE trial [78]. The association we found between attending clinics outside of scheduled visits and PrEP use suggests that the provision of comprehensive prevention services in MSM-friendly sexual health clinics promotes PrEP use. The second finding-the association between often contacting PE outside of scheduled visits and correct adherence-shows that peer education does indeed facilitate correct adherence. At the structural and community-level, we recommend that future PrEP programs ensure MSM have access to friendly clinics where they can go to as often as possible, and where they will not feel ashamed. Furthermore, to aid PrEP users at the individual level, we recommend strengthening the role of PE in PrEP programs especially concerning adherence support.

Both PrEP engagement outcomes also had behavioral type correlates. Specifically, we found PrEP use was linked to CAS and more frequent intercourse with stable partners, while correct adherence was associated with taking a receptive or versatile sexual position with stable partners. Both PrEP use and correct adherence have been associated with engaging in high-risk behaviors [12–15, 48–50]. The prevention-effective adherence paradigm [79] was developed to describe this phenomenon where PrEP users judge their level of risk and adapt their PrEP use and adherence accordingly, in order to ensure HIV protection. It is widely recognized in MSM that condomless receptive anal intercourse has the highest risk of HIV exposure [80], so we can assume that PrEP users would be especially mindful of taking it and adhering correctly to it when engaging in this behavior, but less so for insertive intercourse. This hypothesis was reflected in our results where participants who reported versatile or receptive sexual positions were more likely to report correct adherence. In terms of the differences found between partner types, intercourse with stable partners might be more regular and/or easier to schedule than causal intercourse, so it would be simpler to incorporate PrEP into users’ routines. We recommend incorporating behavioral interventions into PrEP programs to help users develop their ability to accurately evaluate their own HIV risk and adapt their PrEP use to it.

Although the two PrEP engagement outcomes studies here shared certain cohort-related and behavioral characteristics, an important difference existed: the effect of social and economic vulnerabilities was only observed for PrEP adherence. A lack of social and familial support has been shown to impede PrEP adherence for MSM [51–55], especially in low- and middle-income countries [81]. We found that socially isolated participants struggled with adherence as a whole, while those under socioeconomic strain struggled to achieve correct event-driven adherence. It is widely recognized in the literature that event-driven PrEP is harder to adhere to than daily PrEP [45, 65, 69, 70, 82–86]. This finding reinforces prior research by our team on participants in CohMSM-PrEP which found that the compounding nature of socioeconomic strain (i.e., lower socioeconomic status) with event-driven PrEP contributed to ineffective HIV prevention [66]. Structural socioeconomic barriers including unstable housing [6], low income [16] unemployment [9], and transport problems [87] are all associated with low PrEP adherence and/or discontinuation. We assert that for our study sample, these structural factors directly influenced individual health literacy and in turn participants’ ability to understand and adapt to the event-driven PrEP regimen. Although CohMSM-PrEP participants received a comprehensive sexual health package free of charge, as well as transport reimbursement to their study clinic (US$5), these measures seem to have been insufficient in the light of the extreme structural (social, legal, and cultural) barriers for MSM in West Africa. This highlights the need for PrEP programs to directly address socioeconomic barriers to PrEP engagement and to ensure users have a sufficient social support network in place.

It is important to note that the rates of PrEP use and adherence found in the present study (70% and 73%, respectively) were similar to those found in the IPERGAY trial, which tested the efficacy of event-driven PrEP in France and Canada [69, 70]. They reported 71.6% of participants used PrEP during their most recent intercourse and 68.5% of the latter declared correct adherence [88]. However, PrEP use and adherence in the present study were considerably lower than those reported in the PREVENIR cohort, which studied daily and event-driven PrEP roll-out in “real-world” conditions in the Ile-de-France region [89]. Indeed, 85.1% of participants reported PrEP use and 97.5% of them used it correctly [89]. One reason our findings reflect those of IPERGAY more than PREVENIR has to do with the state of PrEP roll-up, which is similar in West Africa now as it was when IPERGAY began in 2015 [69, 70, 90]. Indeed, the PREVENIR cohort exists in a context where PrEP has been widely adopted and there are many resources for PrEP uptake and adherence [89, 90]. Other studies among MSM in a more similar socioeconomic context, i.e. SSA, were found and reported varying degrees of PrEP engagement [48, 86, 91, 92]. For example, among Zimbabwean MSM aware of PrEP, 76.4% of them had used it recently [91]. In terms of correct adherence rates in Kenyan MSM, they varied from 0–14.5% when measured with protective drug concentrations [48, 92] and from 68–83% when measured with a medication event monitoring system [86]. However, these figures are not easily comparable with our study because measurements of PrEP use and adherence were wildly different.

This present study has limitations. First, social desirability bias may have led to over reporting of PrEP outcomes and underreporting of other sensitive topics. To minimize this bias, research assistants were trained to administer all questionnaires during the follow-up period, the assumption being that this repeated and regular contact with participants would help build a trustful relationship over time. Second, the study population comprised a convenience sample of MSM already attending community-based clinics or recruited via peer networks; accordingly, it was not necessarily representative of the local MSM population. However, the differences found in a previous analysis comparing ex-CohMSM participants with newly enrolled participants, suggest that the addition of PrEP to the original project’s (i.e., CohMSM) sexual health prevention offer, and the continued use of peer-based outreach over time helped reach a new profile of MSM less connected to these clinics [64]. Third, the fact that we used only self-reported data to assess PrEP adherence may be considered a less reliable strategy than objective biological measures (plasma, urine, hair, etc.). Nevertheless, multiple studies have shown that drug intake can be accurately predicted by self-reported PrEP outcomes [93–95]. This is especially relevant in low-resource settings, where implementing biological measures is logistically and/or financially complicated [96]. Moreover, in an attempt to preserve the accuracy and objectivity of these self-reported PrEP measures, the CohMSM-PrEP questionnaire was modeled after other cohorts [83, 88, 97] and consequently, our results were easily comparable with other studies and reaffirmed these measures’ utility when studying PrEP adherence [65, 70].

In terms of the study’s strengths, our results contribute to the limited literature on MSM engagement in PrEP in SSA, especially in West Africa where PrEP was only very recently added to a select number of national HIV/AIDS programs. Furthermore, the data from the present study reinforce the relatively little evidence on the positive effect of community-based approaches on PrEP engagement, and advocate the incorporation of PE and the use of MSM-friendly clinics in PrEP implementation in low- and middle-income settings, including West Africa, where MSM are highly stigmatized.

## Conclusions

In a cohort of MSM on PrEP in West Africa, community-based clinic attendance and contact with PE outside of scheduled visits were associated with higher PrEP engagement. However, some socially and economically marginalized participants struggled with adherence. As scale-up continues in West Africa, we recommend implementing community-based interventions and providing extra support for vulnerable PrEP users to ensure adequate PrEP engagement.

## Data Availability

Data can be requested by submitting a study proposal to the scientific board of the CohMSM-PrEP project (christian.laurent@ird.fr). Proposals will be evaluated for compatibility with the CohMSM-PrEP project and overlap with ongoing work. The codes generated during and/or analyzed during the current study are available from the corresponding author on request.

## References

[1] Beach LB, Greene GJ, Lindeman P, Johnson AK, Adames CN, Thomann M (2018). Barriers and Facilitators to Seeking HIV Services in Chicago Among Young Men Who Have Sex with Men: Perspectives of HIV Service Providers. AIDS Patient Care STDs.

[2] Philbin MM, Parker CM, Parker RG, Wilson PA, Garcia J, Hirsch JS (2016). The Promise of Pre-Exposure Prophylaxis for Black Men Who Have Sex with Men: An Ecological Approach to Attitudes, Beliefs, and Barriers. AIDS Patient Care STDs.

[3] Li J, Berg CJ, Kramer MR, Haardörfer R, Zlotorzynska M, Sanchez TH (2019). An Integrated Examination of County- and Individual-Level Factors in Relation to HIV Pre-exposure Prophylaxis Awareness, Willingness to Use, and Uptake Among Men Who Have Sex with Men in the US. AIDS Behav.

[4] Biello KB, Oldenburg CE, Mitty JA, Closson EF, Mayer KH, Safren SA (2017). The “Safe Sex” Conundrum: Anticipated Stigma From Sexual Partners as a Barrier to PrEP Use Among Substance Using MSM Engaging in Transactional Sex. AIDS Behav.

[5] Holloway I, Dougherty R, Gildner J, Beougher SC, Pulsipher C, Montoya JA (2017). PrEP Uptake, Adherence, and Discontinuation among California YMSM Using Geosocial Networking Applications. J Acquir Immune Defic Syndr.

[6] Okafor CN, Gorbach PM, Ragsdale A, Quinn B, Shoptaw S (2017). Correlates of Preexposure Prophylaxis (PrEP) Use among Men Who Have Sex with Men (MSM) in Los Angeles. California J Urban Health.

[7] King HL, Keller SB, Giancola MA, Rodriguez DA, Chau JJ, Young JA (2014). Pre-Exposure Prophylaxis Accessibility Research and Evaluation (PrEPARE Study). AIDS Behav.

[8] Di Ciaccio M, Sagaon-Teyssier L, Protière C, Mimi M, Suzan-Monti M, Meyer L (2019). Impact of HIV risk perception on both pre-exposure prophylaxis and condom use. J Health Psychol.

[9] Whitfield THF, John SA, Rendina HJ, Grov C, Parsons JT (2018). Why I quit pre-exposure prophylaxis (PrEP)? A mixed-method study exploring reasons for PrEP discontinuation and potential re-initiation among gay and bisexual men. AIDS Behav.

[10] Gallagher T, Link L, Ramos M, Bottger E, Aberg J, Daskalakis D (2014). Self-Perception of HIV Risk and Candidacy for Pre-Exposure Prophylaxis Among Men Who Have Sex with Men Testing for HIV at Commercial Sex Venues in New York City. LGBT Health.

[11] Holt M, Murphy DA, Callander D, Ellard J, Rosengarten M, Kippax SC (2012). Willingness to use HIV pre-exposure prophylaxis and the likelihood of decreased condom use are both associated with unprotected anal intercourse and the perceived likelihood of becoming HIV positive among Australian gay and bisexual men. Sex Transm Infect.

[12] Hanum N, Cambiano V, Sewell J, Phillips AN, Rodger AJ, Speakman A (2020). Use of HIV pre-exposure prophylaxis among men who have sex with men in England: data from the AURAH2 prospective study. Lancet Public Health.

[13] Hammoud MA, Vaccher S, Jin F, Bourne A, Maher L, Holt M (2019). HIV Pre-exposure Prophylaxis (PrEP) Uptake Among Gay and Bisexual Men in Australia and Factors Associated With the Nonuse of PrEP Among Eligible Men: Results From a Prospective Cohort Study. J Acquir Immune Defic Syndr.

[14] Gafos M, Horne R, Nutland W, Bell G, Rae C, Wayal S (2019). The Context of Sexual Risk Behaviour Among Men Who Have Sex with Men Seeking PrEP, and the Impact of PrEP on Sexual Behaviour. AIDS Behav.

[15] Liu AY, Cohen SE, Vittinghoff E, Anderson PL, Doblecki-Lewis S, Bacon O (2016). Preexposure Prophylaxis for HIV Infection Integrated With Municipal- and Community-Based Sexual Health Services. JAMA Intern Med.

[16] Wheeler DP, Fields SD, Beauchamp G, Chen YQ, Emel LM, Hightow-Weidman L (2019). Pre-exposure prophylaxis initiation and adherence among Black men who have sex with men (MSM) in three US cities: results from the HPTN 073 study. J Int AIDS Soc.

[17] Kelly JA, Amirkhanian YA, Walsh JL, Brown KD, Quinn KG, Petroll AE (2020). AIDSImpact Special Issue Social Network Intervention to Increase Pre-Exposure Prophylaxis (PrEP) Awareness, Interest, and Use Among African American Men Who Have Sex with Men. AIDS Care.

[18] Eaton LA, Driffin DD, Smith H, Conway-Washington C, White D, Cherry C (2014). Psycho-social factors related to willingness to use pre-exposure prophylaxis for HIV prevention among Black men who have sex with men attending a community event. Sex Health.

[19] Oldenburg CE, Perez-Brumer AG, Hatzenbuehler ML, Krakower D, Novak DS, Mimiaga MJ (2015). State-level structural sexual stigma and HIV prevention in a national online sample of HIV-uninfected men who have sex with men in the United States. AIDS Lond Engl.

[20] Young I, McDaid L (2014). How Acceptable are Antiretrovirals for the Prevention of Sexually Transmitted HIV?: A Review of Research on the Acceptability of Oral Pre-exposure Prophylaxis and Treatment as Prevention. AIDS Behav.

[21] Cáceres CF, Bekker LG, Godfrey-Faussett P (2016). No one left behind: how are we doing in the roll-out of PrEP as part of combination HIV prevention?. J Int AIDS Soc.

[22] Sidebottom D, Ekström AM, Strömdahl S (2018). A systematic review of adherence to oral pre-exposure prophylaxis for HIV – how can we improve uptake and adherence?. BMC Infect Dis.

[23] Golub SA (2018). PrEP Stigma: Implicit and Explicit Drivers of Disparity. Curr HIV/AIDS Rep.

[24] Peng P, Su S, Fairley CK, Chu M, Jiang S, Zhuang X (2018). A Global Estimate of the Acceptability of Pre-exposure Prophylaxis for HIV Among Men Who have Sex with Men: A Systematic Review and Meta-analysis. AIDS Behav.

[25] Ayala G, Makofane K, Santos GM, Beck J, Do TD, Hebert P (2013). Access to Basic HIV-Related Services and PrEP Acceptability among Men Who Have sex with Men Worldwide: Barriers, Facilitators, and Implications for Combination Prevention. J Sex Transm Dis.

[26] UNAIDS (2021). UNAIDS data 2021.

[27] AVAC (2020). Global PrEP Enrollee Tracker.

[28] Djomand G, Quaye S, Sullivan PS (2014). HIV epidemic among key populations in west Africa. Curr Opin HIV AIDS.

[29] Abara WE, Garba I (2017). HIV epidemic and human rights among men who have sex with men in sub-Saharan Africa: Implications for HIV prevention, care, and surveillance. Glob Public Health.

[30] Beyrer C, Baral SD, van Griensven F, Goodreau SM, Chariyalertsak S, Wirtz AL (2012). Global epidemiology of HIV infection in men who have sex with men. Lancet Lond Engl.

[31] Baral S, Scheibe A, Sullivan P, Trapence G, Lambert A, Bekker LG (2013). Assessing priorities for combination HIV prevention research for men who have sex with men (MSM) in Africa. AIDS Behav.

[32] Beyrer C, Baral SD, Collins C, Richardson ET, Sullivan PS, Sanchez J (2016). The global response to HIV in men who have sex with men. The Lancet.

[33] Grosso A, Ryan O, Tram KH, Baral S (2015). Financing the response to HIV among gay men and other men who have sex with men: Case studies from eight diverse countries. Glob Public Health.

[34] Smith AD, Tapsoba P, Peshu N, Sanders EJ, Jaffe HW (2009). Men who have sex with men and HIV/AIDS in sub-Saharan Africa. The Lancet.

[35] Kushwaha S, Lalani Y, Maina G, Ogunbajo A, Wilton L, Agyarko-Poku T (2017). “But the moment they find out that you are MSM…”: a qualitative investigation of HIV prevention experiences among men who have sex with men (MSM) in Ghana’s health care system. BMC Public Health.

[36] Ogunbajo A, Kershaw T, Kushwaha S, Boakye F, Wallace-Atiapah ND, Nelson LE (2018). Barriers, Motivators, and Facilitators to Engagement in HIV Care Among HIV-Infected Ghanaian Men Who have Sex with Men (MSM). AIDS Behav.

[37] Mendos LR (2019). State sponsored homophobia 2019. International Lesbian, Gay, Bisexual, Trans and Intersex Association (ILGA World).

[38] Poteat T, Diouf D, Drame FM, Ndaw M, Traore C, Dhaliwal M (2011). HIV Risk among MSM in Senegal: A Qualitative Rapid Assessment of the Impact of Enforcing Laws That Criminalize Same Sex Practices Kissinger P, editor. PLoS One.

[39] Aho J, Hakim A, Vuylsteke B, Semde G, Gbais HG, Diarrassouba M (2014). Exploring risk behaviors and vulnerability for HIV among men who have sex with men in Abidjan, Cote d’Ivoire: poor knowledge, homophobia and sexual violence. PLoS One.

[40] Duvall S, Irani L, Compaoré C, Sanon P, Bassonon D, Anato S (2015). Assessment of policy and access to HIV prevention, care, and treatment services for men who have sex with men and for sex workers in Burkina Faso and Togo. J Acquir Immune Defic Syndr.

[41] Makofane K, Gueboguo C, Lyons D, Sandfort T (2013). Men who have sex with men inadequately addressed in African AIDS National Strategic Plans. Glob Public Health.

[42] Schwartz SR, Nowak RG, Orazulike I, Keshinro B, Ake J, Kennedy S (2015). The immediate effect of the Same-Sex Marriage Prohibition Act on stigma, discrimination, and engagement on HIV prevention and treatment services in men who have sex with men in Nigeria: analysis of prospective data from the TRUST cohort. Lancet HIV.

[43] Baeten JM, Haberer JE, Liu AY, Sista N (2013). Preexposure Prophylaxis for HIV Prevention: Where Have We Been and Where Are We Going?. JAIDS J Acquir Immune Defic Syndr.

[44] Cáceres CF, Koechlin F, Goicochea P, Sow PS, O’Reilly KR, Mayer KH (2015). The promises and challenges of pre-exposure prophylaxis as part of the emerging paradigm of combination HIV prevention. J Int AIDS Soc.

[45] Grant RM, Lama JR, Anderson PL, McMahan V, Liu AY, Vargas L (2010). Preexposure chemoprophylaxis for HIV prevention in men who have sex with men. N Engl J Med.

[46] Thigpen MC, Kebaabetswe PM, Paxton LA, Smith DK, Rose CE, Segolodi TM (2012). Antiretroviral Preexposure Prophylaxis for Heterosexual HIV Transmission in Botswana. N Engl J Med.

[47] Choopanya K, Martin M, Suntharasamai P, Sangkum U, Mock PA, Leethochawalit M (2013). Antiretroviral prophylaxis for HIV infection in injecting drug users in Bangkok, Thailand (the Bangkok Tenofovir Study): a randomised, double-blind, placebo-controlled phase 3 trial. The Lancet.

[48] Wahome EW, Graham SM, Thiong’o AN, Mohamed K, Oduor T, Gichuru E (2020). PrEP uptake and adherence in relation to HIV-1 incidence among Kenyan men who have sex with men. EClinicalMedicine.

[49] Wahome E, Graham S, Thiong’o A, Chirro O, Mohamed K, Gichuru E (2019). Assessment of PrEP eligibility and uptake among at-risk MSM participating in a HIV-1 vaccine feasibility cohort in coastal Kenya. Wellcome Open Res.

[50] Grant RM, Anderson PL, McMahan V, Liu A, Amico KR, Mehrotra M (2014). Uptake of pre-exposure prophylaxis, sexual practices, and HIV incidence in men and transgender women who have sex with men: a cohort study. Lancet Infect Dis.

[51] Galea JT, Kinsler JJ, Salazar X, Lee SJ, Giron M, Sayles JN (2011). Acceptability of Pre-Exposure Prophylaxis (PrEP) as an HIV prevention strategy: Barriers and facilitators to PrEP uptake among at-risk Peruvian populations. Int J STD AIDS.

[52] Zhang Y, Peng B, She Y, Liang H, Peng HB, Qian HZ (2013). Attitudes Toward HIV Pre-Exposure Prophylaxis Among Men Who Have Sex with Men in Western China. AIDS Patient Care STDs.

[53] Zhou F, Gao L, Li S, Li D, Zhang L, Fan W (2012). Willingness to Accept HIV Pre-Exposure Prophylaxis among Chinese Men Who Have Sex with Men. PLoS One.

[54] Chakrapani V, Newman PA, Shunmugam M, Mengle S, Varghese J, Nelson R (2015). Acceptability of HIV Pre-Exposure Prophylaxis (PrEP) and Implementation Challenges Among Men Who Have Sex with Men in India: A Qualitative Investigation. AIDS Patient Care STDs.

[55] Eisingerich AB, Wheelock A, Gomez GB, Garnett GP, Dybul MR, Piot PK (2012). Attitudes and Acceptance of Oral and Parenteral HIV Preexposure Prophylaxis among Potential User Groups: A Multinational Study. PLoS One.

[56] Tangmunkongvorakul A, Chariyalertsak S, Amico KR, Saokhieo P, Wannalak V, Sangangamsakun T (2013). Facilitators and barriers to medication adherence in an HIV prevention study among men who have sex with men in the iPrEx study in Chiang Mai. Thailand AIDS Care.

[57] Lim SH, Mburu G, Bourne A, Pang J, Wickersham JA, Wei CKT (2017). Willingness to use pre-exposure prophylaxis for HIV prevention among men who have sex with men in Malaysia: Findings from an online survey. PLoS One.

[58] Karuga RN, Njenga SN, Mulwa R, Kilonzo N, Bahati P, O’reilley K (2016). “How I Wish This Thing Was Initiated 100 Years Ago!” Willingness to Take Daily Oral Pre-Exposure Prophylaxis among Men Who Have Sex with Men in Kenya. PLoS One.

[59] Jackson T, Huang A, Chen H, Gao X, Zhong X, Zhang Y (2012). Cognitive, Psychosocial, and Sociodemographic Predictors of Willingness to Use HIV Pre-Exposure Prophylaxis Among Chinese Men Who Have Sex with Men. AIDS Behav.

[60] Schneider JA, Young L, Ramachandran A, Michaels S, Cohen H, Robinson I (2021). A Pragmatic Randomized Controlled Trial to Increase PrEP Uptake for HIV Prevention: 55-Week Results From PrEPChicago. J Acquir Immune Defic Syndr.

[61] McMahan VM, Martin A, Garske L, Violette LR, Andrasik MP, Baeten JM (2019). Development of a targeted educational intervention to increase pre-exposure prophylaxis uptake among cisgender men and transgender individuals who have sex with men and use methamphetamine in Seattle (WA, USA). Sex Health.

[62] Daughtridge GW, Conyngham SC, Ramirez N, Koenig HC (2015). I Am Men’s Health: Generating Adherence to HIV Pre-Exposure Prophylaxis (PrEP) in Young Men of Color Who Have Sex with Men. J Int Assoc Provid AIDS Care JIAPAC.

[63] AVAC (2019). Ongoing and Planned Oral PrEP Studies [Internet]. Ongoing and Planned Oral PrEP Studies.

[64] Eubanks A, Dembélé Keita B, Anoma C, Dah TTE, Mensah E, Maradan G (2020). Reaching a Different Population of MSM in West Africa With the Integration of PrEP Into a Comprehensive Prevention Package (CohMSM-PrEP ANRS 12369-Expertise France). J Acquir Immune Defic Syndr.

[65] Laurent C, Keita BD, Yaya I, Guicher GL, Sagaon-Teyssier L, Agboyibor MK (2021). HIV pre-exposure prophylaxis for men who have sex with men in west Africa: a multicountry demonstration study. Lancet HIV.

[66] Eubanks A, Coulibaly B, Dembélé Keita B, Anoma C, DAH TTE, Mensah E, et al. Rate and Predictors of Ineffective HIV Protection in African Men Who Have Sex with Men Taking Pre-Exposure Prophylaxis [published online ahead of print, 2022 Apr 25]. AIDS Behav. 2022. 10.1007/s10461-022-03692-8. 10.1007/s10461-022-03692-8 35469111

[67] Dah TTE, Yaya I, Sagaon-Teyssier L, Coulibaly A, Kouamé MJB, Agboyibor MK (2021). Adherence to quarterly HIV prevention services and its impact on HIV incidence in men who have sex with men in West Africa (CohMSM ANRS 12324 – Expertise France). BMC Public Health.

[68] Anderson PL, Glidden DV, Liu A, Buchbinder S, Lama JR, Guanira JV (2012). Emtricitabine-tenofovir exposure and pre-exposure prophylaxis efficacy in men who have sex with men. Sci Transl Med.

[69] Molina JM, Capitant C, Spire B, Pialoux G, Cotte L, Charreau I (2015). On-Demand Preexposure Prophylaxis in Men at High Risk for HIV-1 Infection. N Engl J Med.

[70] Molina JM, Charreau I, Spire B, Cotte L, Chas J, Capitant C (2017). Efficacy, safety, and effect on sexual behaviour of on-demand pre-exposure prophylaxis for HIV in men who have sex with men: an observational cohort study. Lancet HIV.

[71] Ha H, Risser JMH, Ross MW, Huynh NT, Nguyen HTM (2015). Homosexuality-related stigma and sexual risk behaviors among men who have sex with men in Hanoi. Vietnam Arch Sex Behav.

[72] Campbell CK, Lippman SA, Moss N, Lightfoot M (2018). Strategies to Increase HIV Testing Among MSM: A Synthesis of the Literature. AIDS Behav.

[73] Dramé FM, Crawford EE, Diouf D, Beyrer C, Baral SD (2013). A pilot cohort study to assess the feasibility of HIV prevention science research among men who have sex with men in Dakar, Senegal. J Int AIDS Soc.

[74] Sullivan PS, Carballo-Diéguez A, Coates T, Goodreau SM, McGowan I, Sanders EJ (2012). Successes and challenges of HIV prevention in men who have sex with men. The Lancet.

[75] Kelly JA, Murphy DA, Sikkema KJ, McAuliffe TL, Roffman RA, Solomon LJ (1997). Randomised, controlled, community-level HIV-prevention intervention for sexual-risk behaviour among homosexual men in US cities. The Lancet.

[76] Amirkhanian YA, Kelly JA, Kabakchieva E, Kirsanova AV, Vassileva S, Takacs J (2005). A randomized social network HIV prevention trial with young men who have sex with men in Russia and Bulgaria. AIDS.

[77] Kelly JA, Amirkhanian YA, Kabakchieva E, Vassileva S, Vassilev B, McAuliffe TL (2006). Prevention of HIV and sexually transmitted diseases in high risk social networks of young Roma (Gypsy) men in Bulgaria: randomised controlled trial. BMJ.

[78] Mehrotra ML, Amico KR, McMahan V, Glidden DV, Defechereux P, Guanira JV (2018). The role of social relationships in PrEP uptake and use among transgender women and men who have sex with men. AIDS Behav.

[79] Haberer JE, Bangsberg DR, Baeten JM, Curran K, Koechlin F, Amico KR (2015). Defining success with HIV pre-exposure prophylaxis: A prevention-effective adherence paradigm. AIDS Lond Engl.

[80] Patel P, Borkowf CB, Brooks JT, Lasry A, Lansky A, Mermin J (2014). Estimating per-act HIV transmission risk: a systematic review. AIDS.

[81] Yi S, Tuot S, Mwai GW, Ngin C, Chhim K, Pal K (2017). Awareness and willingness to use HIV pre-exposure prophylaxis among men who have sex with men in low- and middle-income countries: a systematic review and meta-analysis. J Int AIDS Soc.

[82] Van der Elst EM, Mbogua J, Operario D, Mutua G, Kuo C, Mugo P (2013). High Acceptability of HIV Pre-exposure Prophylaxis but Challenges in Adherence and Use: Qualitative Insights from a Phase I Trial of Intermittent and Daily PrEP in At-Risk Populations in Kenya. AIDS Behav.

[83] Di Ciaccio M, Sagaon-Teyssier L, Mimi M, Suzan-Monti M, Protiere C, Rojas Castro D, et al. Changes in sexual behaviors in men who have sex with men: a comparison between the double-blind and open-label extension phases of the ANRS-IPERGAY trial. AIDS Behav. 2020;24(11):3093–106. 10.1007/s10461-020-02864-8 32306213

[84] Gafos M, White D, Clarke A, Apea V, Brodnicki E, Mackie N, et al. Adherence intentions, long-term adherence and HIV acquisition among PrEP users in the PROUD open-label randomised control trial of PrEP in England. 9th IAS conference on HIV Science, July 23-26, 2017, Paris.

[85] Hosek SG, Siberry G, Bell M, Lally M, Kapogiannis B, Green K (2013). The acceptability and feasibility of an HIV preexposure prophylaxis (PrEP) trial with young men who have sex with men. J Acquir Immune Defic Syndr.

[86] Mutua G, Sanders E, Mugo P, Anzala O, Haberer JE, Bangsberg D (2012). Safety and Adherence to Intermittent Pre-Exposure Prophylaxis (PrEP) for HIV-1 in African Men Who Have Sex with Men and Female Sex Workers. Maartens G, editor. PLoS One.

[87] Morgan E, Ryan DT, Newcomb ME, Mustanski B (2018). High Rate of Discontinuation May Diminish PrEP Coverage Among Young Men Who Have Sex with Men. AIDS Behav.

[88] Sagaon-Teyssier L, Suzan-Monti M, Demoulin B, Capitant C, Lorente N, Préau M (2016). Uptake of PrEP and condom and sexual risk behavior among MSM during the ANRS IPERGAY trial. AIDS Care.

[89] Molina JM, Ghosn J, Assoumou L, Delaugerre C, Algarte-Genin M, Pialoux G, et al. Daily and on-demand HIV pre-exposure prophylaxis with emtricitabine and tenofovir disoproxil (ANRS PREVENIR): a prospective observational cohort study. Lancet HIV. 2022;9(8):e554–62. 10.1016/S2352-3018(22)00133-3 35772417

[90] AVAC (2022). PrEPWatch [Internet]. PrEPWatch.

[91] Parmley LE, Harris TG, Chingombe I, Mapingure M, Mugurungi O, Rogers JH (2022). Engagement in the pre-exposure prophylaxis (PrEP) cascade among a respondent-driven sample of sexually active men who have sex with men and transgender women during early PrEP implementation in Zimbabwe. J Int AIDS Soc.

[92] Kimani M, van der Elst EM, Chirro O, Wahome E, Ibrahim F, Mukuria N (2021). “I wish to remain HIV negative”: Pre-exposure prophylaxis adherence and persistence in transgender women and men who have sex with men in coastal Kenya. PLoS One.

[93] Amico KR, Mehrotra M, Avelino-Silva VI, McMahan V, Veloso VG, For the iPrEx Study Team (2016). Self-reported Recent PrEP Dosing and Drug Detection in an Open Label PrEP Study. AIDS Behav.

[94] Blumenthal J, Pasipanodya EC, Jain S, Sun S, Ellorin E, Morris S (2019). Comparing Self-Report Pre-Exposure Prophylaxis Adherence Questions to Pharmacologic Measures of Recent and Cumulative Pre-Exposure Prophylaxis Exposure. Front Pharmacol.

[95] Marins LMS, Torres TS, Leite I da C, Moreira RI, Luz PM, Hoagland B (2019). Performance of HIV pre-exposure prophylaxis indirect adherence measures among men who have sex with men and transgender women: Results from the PrEP Brasil Study. Patel RR, editor. PLOS ONE.

[96] Haberer JE (2016). Current concepts for PrEP adherence in the PrEP revolution: from clinical trials to routine practice. Curr Opin HIV AIDS.

[97] Sagaon-Teyssier L, Mabire X, Laguette V, Fressard L, Suzan-Monti M, Rojas Castro D (2018). A Group-Based Trajectory Model for Changes in Pre-Exposure Prophylaxis and Condom Use Among Men Who Have Sex with Men Participating in the ANRS IPERGAY Trial. AIDS Patient Care STDs.

